# International Consortium for Health Outcomes Measurement (ICHOM)

**DOI:** 10.1186/1745-6215-16-S3-O4

**Published:** 2015-11-24

**Authors:** Thomas A Kelley

**Affiliations:** 1 International Consortium for Health Outcomes Measurement, London, UK

## Introduction

Outcomes that reflect what matter most to patients are infrequently measured in clinical practice. When they are captured, the definitions used often vary between countries, making global comparisons and learning impossible. A similar challenge exists in clinical trial endpoints.

## Methods

ICHOM was founded in 2012 to address these challenges. ICHOM brings together working groups, organised around the medical condition, consisting of patients, health professionals, researchers, outcomes measurement experts and policy makers, from all major regions of the world. Working groups follow a structured series of teleconferences, facilitated by ICHOM, with each teleconference covering a set topic, followed by a survey for working group members to complete. The end result is a globally agreed set of outcomes that reflects what matters most to most patients. ICHOM sets aim to be used in both routine clinical practice and as an endpoint in clinical studies.

## Results

ICHOM has now produced 12 standard sets of outcomes covering approximately 35% of the global burden of disease. These sets include: cataracts; localised and advanced prostate cancer; lower back pain; coronary artery disease; Parkinson’s disease; cleft lip and palate; stroke; macular degeneration; hip and knee osteoarthritis; depression and anxiety; lung cancer. ICHOM is now forming networks of hospitals around the world, which are working together to begin measuring, benchmarking, and performing outcome comparisons and translating this into subsequent learning.

## Conclusion

ICHOM is working with health systems and measurement bodies all over the world to produce and implement, in clinical practice and in clinical studies, globally agreed standard sets of outcomes that reflect what matter most to patients. This will enable global outcome comparisons and translatable learning.

